# Exploring factors influencing asthma control and asthma-specific health-related quality of life among children

**DOI:** 10.1186/1465-9921-14-26

**Published:** 2013-02-23

**Authors:** Pranav K Gandhi, Kelly M Kenzik, Lindsay A Thompson, Darren A DeWalt, Dennis A Revicki, Elizabeth A Shenkman, I-Chan Huang

**Affiliations:** 1 Department of Pharmacy Practice, School of Pharmacy, South College, Knoxville, TN, USA; 2 Department of Health Outcomes and Policy, College of Medicine, University of Florida, Gainesville, FL, USA; 3 Department of Pediatrics, College of Medicine, University of Florida, Gainesville, FL, USA; 4 Department of Medicine, School of Medicine, University of North Carolina-Chapel Hill, Chapel Hill, NC, USA; 5 Outcomes Research, United BioSource Corporation, Bethesda, MD, USA; 6 Institute for Child Health Policy, College of Medicine, University of Florida, Gainesville, FL, USA

**Keywords:** Asthma control, Health-related quality of life, PROMIS, Satisfaction with shared decision-making, Perceived self-efficacy with patient-physician interaction, Structural equation modeling

## Abstract

**Background:**

Little is known about factors contributing to children’s asthma control status and health-related quality of life (HRQoL). The study objectives were to assess the relationship between asthma control and asthma-specific HRQoL in asthmatic children, and to examine the extent to which parental health literacy, perceived self-efficacy with patient-physician interaction, and satisfaction with shared decision-making (SDM) contribute to children’s asthma control and asthma-specific HRQoL.

**Methods:**

This cross-sectional study utilized data collected from a sample of asthmatic children (n = 160) aged 8–17 years and their parents (n = 160) who visited a university medical center. Asthma-specific HRQoL was self-reported by children using the National Institutes of Health’s Patient-Reported Outcomes Measurement Information System (PROMIS) Pediatric Asthma Impact Scale. Satisfaction with SDM, perceived self-efficacy with patient-physician interaction, parental health literacy, and asthma control were reported by parents using standardized measures. Structural equation modeling (SEM) was performed to test the hypothesized pathways.

**Results:**

Path analysis revealed that children with better asthma control reported higher asthma-specific HRQoL (β = 0.4, *P* < 0.001). Parents with higher health literacy and greater perceived self-efficacy with patient-physician interactions were associated with higher satisfaction with SDM (β = 0.38, *P* < 0.05; β = 0.58, *P* < 0.001, respectively). Greater satisfaction with SDM was in turn associated with better asthma control (β = −0.26, *P* < 0.01).

**Conclusion:**

Children’s asthma control status influenced their asthma-specific HRQoL. However, parental factors such as perceived self-efficacy with patient-physician interaction and satisfaction with shared decision-making indirectly influenced children’s asthma control status and asthma-specific HRQoL.

## Introduction

Asthma is a chronic disease caused by inflammation of the airways that leads to narrowed airways or bronchoconstriction [1]. Children aged 0 – 17 years have higher asthma prevalence (9.6%) compared with adults (7.7%) in the US [2]. Previous studies have showed that among children with asthma the prevalence of poorly-controlled asthma status varied (32% - 64%) [3-5]. For example, one study reported 46% of the asthmatic children who used inhaled corticosteroids had poorly-controlled asthma [5]. Inadequate asthma control causes an increase in the frequency and sometimes the severity of asthma attacks [6,7]. Asthma control is a multidimensional concept which is defined as “patient reports of daytime and nocturnal symptoms, activity limitations due to asthma, need for rescue medications, and measures of lung function [8].” The Asthma Guidelines of the National Asthma Education and Prevention Program (NAEPP) published by the National Institutes of Health (NIH) emphasize the need to evaluate asthma control as a key component for asthma treatment and management [8-11].

Well-controlled asthma is associated with improved health status [12], and fewer physician visits, hospitalizations and emergency room visits among children and adults [11,13]. In contrast, poor asthma control is directly linked with increased school absenteeism and loss in work productivity among asthma children and adults/caregivers, respectively [14,15]. Unfortunately, studies report that asthma remains uncontrolled in many asthmatic patients, despite receiving appropriate treatments [16]. Patient-reported outcomes, such as health-related quality of life (HRQoL), are useful indicators to understand the impact of poor asthma control on functional status and well-being [17]. Several studies have investigated the impact of asthma control on asthma-specific and generic HRQoL, where poorly controlled asthma was found to be associated with lower HRQoL scores [13-15]. The most recent NAEPP guidelines (2007 update) emphasize the need to investigate the impact of asthma control on HRQoL [9], especially since clinicians do not strictly follow the NAEPP guidelines to address asthma control status [14,18].

Factors influencing asthma control, which in turn affect HRQoL, are complex and undetermined [8,17]. Previous studies have consistently identified that individual factors such as genetics, smoking, poor design of inhaler device, improper medication compliance, as well as family and environmental factors such as pets in the home, air pollution, and pollen exposure are important determinants of poorly controlled asthma [9,19,20]. Recently, more attention has been focused on the impact of parental factors on asthma outcomes. Parental health literacy of children with asthma, for example, is one such factor that might directly contribute to the optimal asthma care of their children [21]. Low health literacy levels could influence parents’ understanding of asthma etiology and impact capability of engaging in the decision-making process with providers, and compliance with the treatment plan. The NAEPP guidelines emphasize that physicians should engage children and parents in the decision-making process and assessment of asthma control for effective asthma management [9]. Higher health literacy was associated with higher perceived self-efficacy in asthma management or a greater desire to actively engage in the decision-making process [22,23], whereas another study could not replicate these findings [24]. In addition, several studies have shown parental factors such as health literacy, self-efficacy or satisfaction with the decision-making process to be significantly associated with asthma outcomes, including HRQoL [25-27]. Nevertheless, limited evidence is available examining the complex relationships among parental health literacy, perceived self-efficacy with patient-physician interaction, and satisfaction with shared decision-making (SDM), especially the impact of these factors on pediatric asthma outcomes.

The present study aimed to assess the relationship between asthma control and HRQoL in children with asthma. We anticipated that in contrast to children with good asthma control, children with poor asthma control would report poorer asthma-specific HRQoL. Second, we aimed to examine how parental health literacy, perceived self-efficacy with patient-physician interaction, and satisfaction with SDM contributed to children’s asthma control and asthma-specific HRQoL. We hypothesized that greater parental health literacy and greater perceived self-efficacy would be significantly associated with higher satisfaction with SDM, and in turn higher satisfaction with SDM would be associated with better pediatric asthma control status and asthma-specific HRQoL. Specifically, we used structural equation modeling (SEM) to simultaneously analyze the complex relationships among the aforementioned variables. The present study will extend the literature to demonstrate important factors that contribute to pediatric asthma control and asthma-specific HRQoL.

## Methods

### Data sources, data collection, and study sample

This is a cross-sectional study using data collected from a sample of asthmatic children (n = 160) aged 8–17 years and their parents/guardians (n = 160) who visited five pediatric clinics within the University of Florida (UF) Health Science Center in Gainesville, Florida. The clinics include Pediatric Primary Care at Gerold L. Schiebler CMS Center and Tower Square, Pediatric After Hours Clinic, Pediatric Allergy Clinic, and Pediatric Pulmonary Clinic. During the clinical appointment, physicians of the five pediatric clinics identified eligible participants based on the following enrollment criteria: age range, symptoms and medication use as indicated in the medical record, and fluency level in English language. Physicians used the asthmatic symptoms and medication use as suggested in NAEPP asthma guideline to determine asthma status [9]. Eligible participants were subsequently referred to research assistants who were in the waiting room area. Research assistants guided the parents to complete written informed consent form (and children needed to assent), followed by a completion of survey questionnaires in a quiet room of the clinic. We did not ask physicians to count the number of eligible subjects who declined to participate in part due to the busy schedule in clinics. Data were collected between April 2010 and September 2011. UF’s Institutional Review Board approved the study protocol.

### Measurements in survey

A survey questionnaire comprised of different measures was administered septely to children and their parents. Specifically, children answered the items measuring asthma-specific HRQoL, and parents answered items measuring health literacy, self-efficacy with patient-physician interaction, satisfaction with SDM, and asthma control.

#### Asthma-specific HRQoL

Asthma-specific HRQoL scale is one of the NIH’s Patient-Reported Outcomes Measurement Information System (PROMIS) pediatric scales that are designed to measure important domains of pediatric patient-reported outcomes [28]. The present study used the asthma impact domain (8 items) to measure asthma-specific HRQOL in children. The response categories for each of the 8 items are never, almost never, sometimes, often and almost always. Each item asks the children themselves about asthma relevant symptoms in the past seven days before the interview. The domain score was calculated by item response theory (IRT), with a mean of 50 and SD of 10, and higher scores for greater impairment in HRQOL. The scale has demonstrated high measurement precision and construct validity based on IRT [28].

#### Asthma control

Asthma control was measured using a 10-item parental questionnaire, which was developed based on the NAEPP Asthma Guidelines published by the NIH [29]. The parents of children with asthma were asked about how often in the past seven days his/her child being bothered by asthma symptoms, number of days for asthma symptoms, use of rescue medications, number of days child with an asthma attack, activity limitations due of asthma, and his/her child being woken by asthma. The asthma control questionnaire has demonstrated good psychometric properties, including internal consistency reliability, convergent/discriminant validity, and known-groups validity [29]. Each item in the asthma control domain is dichotomized as no control problem versus a control problem, where intermittent status is considered as controlled and persistent status is considered as not controlled. For each patient, the values of the five items are summarized to generate an index ranging from 0 to 5, with 0–1 indicating “good control” and 2–5 indicating “poor control.”

#### Health literacy

Parental health literacy was measured using the Short Test of Functional Health Literacy in Adults (S-TOFHLA) [30]. The instrument has demonstrated excellent psychometric properties, including internal consistency reliability (Cronbach’s alpha = 0.98) and test-retest reliability. S-TOFHLA consists of 36 items where each item is dichotomized and scored as “1” for correct and “0” for incorrect. A summed overall score (range: 0–36) for health literacy items is calculated, where higher scores indicate greater health literacy. S-TOFHLA scale is divided into three categories of functional literacy: inadequate (0–16), adequate (17–22), and functional (23–36). Based on the scoring guideline, we classified parents with scores <23 as inadequate or marginal functional health literacy, whereas those with scores ≥23 as adequate functional health literacy [30].

#### Perceived self-efficacy with patient-physician interaction

Perceived self-efficacy with patient-physician interaction (PEPPI) was measured using a standardized scale comprised of 10 items [31]. A five-point response category for each item was utilized (from “not at all” to “very much”). The total scores for the PEPPI scale range from 0 to 50. Higher item and scale scores indicate greater perceived self-efficacy with patient-physician interaction. The scale has demonstrated high internal consistency reliability (Cronbach’s alpha = 0.91) and convergent and discriminant validity [31].

#### Satisfaction with SDM

Satisfaction with shared decision-making for parents was measured using a standardized scale comprised of nine items [32]. A six-point response category for each item was utilized (from “completely disagree” to “completely agree”). A summed raw total score ranging from 0 to 45 was created. Higher item and higher scale scores indicate greater satisfaction with shared decision-making. The scale has demonstrated excellent internal consistency reliability with a Cronbach’s alpha of 0.94, as well as acceptable face validity [32].

### Statistical analysis

Descriptive analyses, including means and standard deviations, were performed to document the characteristics of children and their parents. Pearson’s correlation coefficient was calculated to demonstrate the magnitude of the association between the variables (i.e., asthma-specific HRQoL, asthma control, perceived self-efficacy with patient-physician interaction, satisfaction with SDM, and parental health literacy).

LISREL 8.8 [33] was used to perform SEM and SAS 9.1 software [34] was used for the remaining analyses. Conventionally, regression analysis has been used to examine the relationship between each dependent variable and one or more independent variables. In SEM, a variable may serve as an independent, mediating, or dependent variable depending upon the specific role the variable plays. One variable serving as a dependent variable in one regression model can serve as an independent or mediating variable in other regression models. For example, in one regression model, asthma control is the mediating variable between satisfaction with shared decision-making (independent variable) and asthma-specific HRQOL (dependent variable), whereas in another regression model, asthma control is the dependent variable that is influenced by health literacy (independent variable) and satisfaction with shared decision-making (mediating variable). A variable is qualified as a mediator if the mediating role is significant in path analyses (e.g., an independent variable is significantly associated with a mediating variable and this mediating variable is significantly associated with dependent variable) and the total effect of this path analysis is significant as well. Another unique feature of the SEM application is allowing the simultaneous testing of the measurement and the structural parts in the same analytic framework. The measurement part builds the relationships between the concept of interest (e.g., HRQoL) and indicators (i.e., items) designed to measure that specific concept. The structural part builds the relationship between the variables of interest that were pre-specified in our conceptual framework [35,36].

The selection of variables into the path analyses is based on evidence from the literature, our conceptual framework, and results of bivariate analyses. We did not solely rely on the results of bivariate analyses to guide the path analyses because bivariate analyses do not account for the influence of confounding variables on the relationships among independent, mediating, and dependent variables. In the measurement part of the SEM, we treated self-efficacy with patient-physician interaction, satisfaction with SDM, asthma control and asthma-specific HRQoL as latent variables, which were indirectly measured using the items of instruments. We also calculated internal consistency (Cronbach’s alpha) for each latent variable, with a value of ≥0.7 deemed satisfactory.

The structural part of the SEM involves a path analysis to estimate regression coefficients representing the direct relationships between variables of interests. In addition, SEM allows analyzing the indirect effects of independent variables on dependent variables through the effects of mediating variables. Mediating effects show the impact of a predictor variable on the specific variable of interest which is partially or completely explained by another variable. As aforementioned, path analyses are the extension of regression models, and different covariates that confound the relationships among independent, mediating, and dependent variables can be adjusted. Based on the literature, parents’ age and gender, children’s age and gender, and physician’s report of pediatric comorbid conditions are important covariates; therefore, we included these variables as covariates in the path analyses. We treated health literacy as an independent variable; however, we regarded health literacy as an observed rather than a latent variable because the *meaningful cutoffs* for different levels of health literacy which have been established by the developers were estimated based on the observed scores.

Indices of model fit were estimated to examine the appropriateness of the SEM, including the goodness-of-fit chi-square (*χ*^2^) and Root Mean Square Error of Approximation (RMSEA). A value below 0.08 on RMSEA is deemed a good model fit and a value below 0.05 or less is deemed a close fit [37].

## Results

### Characteristics of the study sample

Table 1 shows the characteristics of children with asthma (n = 160) and their parents (n = 160). Children’s age ranged from 8 to 17 years (mean 11.61 years; SD 2.41 years). Majority of the children in the sample were females (n = 95), and had ≥1 comorbid condition (n = 103). Approximately half of the children had good (n = 77) and poor (n = 83) asthma control. Parents’ age ranged from 25 to 68 years (mean 40.10 years; SD 9.65 years), and they were predominantly females (n = 146), Blacks (n = 87), and had at least some college or associate degree (n = 102). Most of the parents (n = 150) had adequate functional health literacy. Body mass index (BMI) was calculated as the weight in kilograms divided by the height in meters squared. Growth reference charts developed by the World Health Organization BMI were used to categorize each child into different weight categories. More than half of the children (n = 86) were overweight/obese.

**Table 1:** Sample characteristics

**Variables**	**Mean (SD)**
Children characteristics	
Age (in years)	11.61 (2.41)
Gender	
Male	59.38%
Female	40.63%
Race	
White, non-Hispanic	31.88%
Black, non-Hispanic	55.63%
Hispanic/other	12.50%
Asthma control status	
Good control	48.13%
Poor control	51.88%
Baseline comorbid conditions	
Zero comorbid condition	35.63%
> = 1 comorbid condition	64.38%
Specific conditions	
Atopic disease	37.5%
Attention deficit hyperactivity disorder/other learning disability	10.63%
Mental health condition	3.13%
High blood pressure	1.88%
Thyroid disease	1.25%
Born prematurely	1.25%
Other	14.38%
Body Mass Index	
Severe thinness/thinness	2.5%
Normal	34.38%
Overweight/obese	53.75%
Parent characteristics	
Age (in years)	40.10 (9.65)
Gender	
Male	8.75%
Female	91.25%
Race	
White, non-Hispanic	38.75%
Black, non-Hispanic	54.38%
Hispanic/other	6.88%
Education	
Less than high school	14.38%
High school/general educational development	21.88%
Some college/associate degree	41.88%
College degree/some grad school	21.88%
Family income^#^	
Less than 10,000	20.00%
10,000 – 29,999	37.50%
30,000 – 59,999	24.38%
> $60,000	15.63%
Health literacy	
< = 22 (inadequate or marginal functional)	6.26%
> = 23 (adequate functional)	93.75%

SD: standard deviation.

^#^ Due to missing data, these values may not add up to 100%.

### Correlations among variables of interest included in the model

Table 2 shows the bivariate correlations between the variables of interests. The strongest relationship was found between the perceived self-efficacy with patient-physician interaction and satisfaction with SDM (r = 0.59, *P* < 0.001), where parents that reported greater perceived self-efficacy were more likely to report greater satisfaction with SDM than parents with less perceived self-efficacy. Parental health literacy was significantly associated with satisfaction with SDM, yet the magnitude was small (r = 0.19, *P* < 0.05). Satisfaction with SDM was significantly associated with asthma control (r = −0.22, *P* < 0.01), where parents with greater satisfaction with SDM were less likely to report poor asthma control in children. Asthma control was significantly associated with asthma-specific HRQoL (r = 0.40, *P* < 0.001). Children with well controlled status reported better asthma-specific HRQoL compared to children with poorly controlled status.

**Table 2:** Bivariate correlations among variables of interests

**Variable**	**Health literacy**	**Perceived self-efficacy**	**Satisfaction with shared decision-making**	**Asthma control**	**Asthma-specific HRQoL**
Health literacy	1	-	-	-	-
Perceived self-efficacy	0.02	1	-	-	-
Satisfaction with shared decision-making	0.19*	0.59***	1	-	-
Asthma control	−0.06	−0.15	−0.22**	1	-
Asthma-specific HRQoL	−0.10	−0.07	−0.10	0.40***	1

HRQoL: health-related quality of life.

*p < 0.05; **p < 0.01; ***p < 0.001.

### Measurement model

Table 3 shows the measurement part of the SEM, including internal consistent reliability (Cronbach’s alpha) of the four latent variables, and factor loadings (λ) for items associated with the latent variables. In general, all items were significantly associated with corresponding latent variables with acceptable levels of factor loadings (λ > 0.4; *P* < 0.001). The range of factor loadings was 0.46-0.83 for perceived self-efficacy, 0.53-0.86 for satisfaction with SDM, 0.67-0.82 for asthma control, and 0.53-0.79 for asthma-specific HRQoL. These results were consistent with the finding of Cronbach’s alpha, where the values were 0.91 for perceived self-efficacy with patient-physician interaction, 0.94 for satisfaction with SDM, 0.84 for asthma control, and 0.87 for asthma-specific HRQoL.

**Table 3:** Measurement model for latent factors and indicator variables

**Latent factor and indicator variables**	**Response scale**	**Mean (SD)**	Factor loading, λ***
Perceived self-efficacy			
Cronbach’s alpha: 0.91			
1. Confident in ability to get a Dr. to pay attention to what you say	1–5^a^	4.44 (0.81)	0.71
2. Confident in ability to know what questions to ask	1–5^a^	4.29 (0.89)	0.60
3. Confident in ability to get a Dr. to answer all questions	1–5^a^	4.31 (0.78)	0.79
4. Confident in ability to ask Dr. questions about chief concern	1–5^a^	4.50 (0.73)	0.77
5. Confident in ability to make most of visit with Dr.	1–5^a^	4.44 (0.77)	0.80
6. Confident in ability to get Dr. take chief concern seriously	1–5^a^	4.44 (0.77)	0.83
7. Confident in ability to understand what Dr. tells you	1–5^a^	4.56 (0.64)	0.46
8. Confident in ability to get a Dr. to do something about concern	1–5^a^	4.44 (0.69)	0.83
1. Confident in ability to explain chief health concern to Dr.	1–5^a^	4.43 (0.77)	0.63
2. Confident in ability to ask Dr. for more information	1–5^a^	4.54 (0.64)	0.67
Satisfaction with shared decision-making			
Cronbach’s alpha: 0.94			
1. Dr. made it clear that a decision needs to be made	1–6^b^	5.04 (1.12)	0.53
2. Dr. wanted to know exactly how I wanted to be involved	1–6^b^	5.01 (1.09)	0.74
3. Dr. told me there are different option for treatment	1–6^b^	4.86 (1.29)	0.70
4. Dr. explained adv/disadv of treatment options	1–6^b^	4.94 (1.18)	0.85
5. Dr. helped me to understand all info	1–6^b^	5.18 (1.02)	0.78
6. Dr. asked me what option I prefer	1–6^b^	4.81 (1.32)	0.80
7. Dr. and I weight options	1–6^b^	4.75 (1.37)	0.84
8. Dr. and I selected a treatment option together	1–6^b^	4.68 (1.44)	0.86
9. Dr. and I reached an agreement on how to proceed	1–6^b^	5.05 (1.08)	0.86
Asthma control			
Cronbach’s alpha: 0.84			
1. Recode of item: number of days child has symptoms	1–4^c^	1.49 (0.87)	0.67
2. Recode of item: number of days child had to use rescue asthma medicine	1–4^c^	1.31 (0.65)	0.71
3. Recode of item: number of days child had an asthma attack	1–4^c^	1.58 (0.90)	0.82
4. Recode of item: how much did child’s asthma limit activities	1–4^c^	1.40 (0.75)	0.72
5. Recode of item: how many nights did asthma wake him/her up	1–3^d^	1.28 (0.54)	0.71
Asthma-specific HRQoL			
Cronbach’s alpha: 0.87			
3. Felt scared because of breathing	1–5^e^	2.35 (1.13)	0.53
4. Chest felt tight because of asthma	1–5^e^	2.50 (1.26)	0.70
5. Felt wheezy	1–5^e^	2.99 (1.27)	0.73
6. Trouble breathing	1–5^e^	3.07 (1.26)	0.79
7. Trouble sleeping	1–5^e^	2.42 (1.22)	0.59
8. Hard to play sports or exercise	1–5^e^	2.79 (1.43)	0.65
9. Hard to take a deep breath	1–5^e^	2.48 (1.24)	0.68
10. Asthma bothered me	1–5^e^	2.95 (1.37)	0.73

SD: standard deviation; HRQoL: health-related quality of life.

***p < 0.001.

^a^ (1) Not at all; (5) Very much.

^b^ (1) Completely disagree; (6) Completely agree.

^c^ (1) Mild intermittent; (4) Severe persistent.

^d^ (1) Mild intermittent; (3) Moderate persistent.

^e^ (1) Never; (5) Almost always.

### Path analysis for relationships among variables included in the structural model

**Figure 1:**
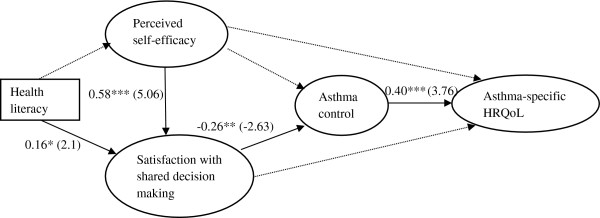
**Path analysis for the relationships between health literacy, perceived self-efficacy, satisfaction with shared decision-making, asthma control, and asthma-specific HRQoL.** HRQoL: health-related quality of life. Dotted lines indicate statistically non-significant pathways and solid lines indicate statistically significant pathways. Values represent standardized pmeter estimates and t-values (in parentheses). Model fit for the model includes solid lines only: *χ*^2^ (degrees of freedom): 1036.69 (624), and RMSEA (90% CI): 0.064 (0.057 – 0.071). *p < 0.05; **p < 0.01; ***p < 0.001.

We first tested the full path analytic model which includes all the hypothesized variables. The fit of the full model was deemed satisfactory although the relationships between some variables were not statistically significant (*P* > 0.05). The paths from health literacy to perceived self-efficacy with patient-physician interaction, from perceived self-efficacy with patient-physician interaction to asthma control and asthma-specific HRQoL, and from satisfaction with SDM to asthma-specific HRQoL were not statistically significant (detailed results available upon request). Figure 1 shows the reduced path analytic model with standardized path coefficients that were statistically significant (*P* < 0.05). Compared to the full model, the reduced model improved the model fit slightly (*χ*^2^ (df) = 1036.69 (624); RMSEA (90% CI) = 0.064 (0.057 – 0.071)).

Table 4 shows the direct and indirect effects among variable of interests derived from the reduced path analytic model shown in Figure 1. Asthma-specific HRQoL was significantly associated with asthma control status, where children with good asthma control status reported higher asthma-specific HRQoL compared to those with poor asthma control (β = 0.4, *P* < 0.001). Parents with higher health literacy and greater perceived self-efficacy with patient-physician interaction had higher satisfaction with SDM (β = 0.38, *P* < 0.05; β = 0.58, *P* < 0.001, respectively). However, greater perceived self-efficacy with patient-physician interaction was indirectly associated with higher asthma-specific HRQoL through greater satisfaction with SDM and better asthma control (β = −0.06, *P* < 0.05). In addition, greater perceived self-efficacy with patient-physician interaction was indirectly associated with well-controlled asthma through greater satisfaction with SDM (β = −0.15, *P* < 0.01). Greater satisfaction with SDM was significantly associated with asthma control, where parents with higher satisfaction with SDM reported well controlled asthma compared with those with lower satisfaction with SDM (β = −0.26, *P* < 0.01). Finally, greater satisfaction with SDM was indirectly associated with higher asthma-specific HRQoL through better asthma control (β = −0.11, *P* < 0.05).

**Table 4:** Direct, indirect, and total effects among variables of interests

**Latent variables**	**Standardized pmeter estimates and standard error (in parentheses)**
Direct effects	
Health literacy → satisfaction with shared decision-making	0.38* (0.18)
Perceived self-efficacy → satisfaction with shared decision-making	0.58*** (0.12)
Satisfaction with shared decision-making → asthma control	−0.26** (0.10)
Asthma control → asthma-specific HRQoL	0.40*** (0.11)
Indirect effects	
Perceived self-efficacy → satisfaction with shared decision-making → asthma control	−0.15** (0.06)
Perceived self-efficacy → Satisfaction with shared decision-making → asthma control → asthma-specific HRQoL	−0.06** (0.03)
Satisfaction with shared decision-making → asthma control → asthma-specific HRQoL	−0.11* (0.05)
Total effects	
Health literacy → perceived self-efficacy → satisfaction with shared decision-making	0.48* (0.21)
Perceived self-efficacy → satisfaction with shared decision-making	0.58*** (0.12)
Perceived self-efficacy → satisfaction with shared decision-making → asthma control	−0.15** (0.06)
Satisfaction with shared decision-making → asthma control	−0.26** (0.10)
Perceived self-efficacy → Satisfaction with shared decision-making → asthma control → asthma-specific HRQoL	−0.06* (0.03)
Satisfaction with shared decision-making → asthma control → asthma-specific HRQoL	−0.11* (0.05)
Asthma control → asthma-specific HRQoL	0.40*** (0.11)

HRQoL: health-related quality of life.

Values represent standardized pmeter estimates and standard error (in parentheses).

*p < 0.05; **p < 0.01; ***p < 0.001.

## Discussion

The primary goal of asthma treatment emphasized in the national and international asthma guidelines is to control asthma symptoms and prevent asthma flares making it imperative to evaluate asthma control status for every patient [9,10]. Our results, reflecting and expanding previous studies [13-15], indicate that good asthma control status is associated with better HRQoL. While previous work has examined the association between asthma control and HRQoL in children [15,38], these studies did not explicitly test which parental factors influence asthma control status, in turn affecting asthma-specific HRQoL. The present study shows how the parental factors such as health literacy, satisfaction with SDM, and perceived self-efficacy with patient-physician interaction directly and indirectly affect asthma control through different pathways, which in turn influences pediatric asthma-specific HRQoL. Notably, the association between asthma control and asthma-specific HRQoL remained strong after taking into account the influence of these factors. Understanding and accounting for these factors may help practitioners identify patients at an increased risk of poor asthma control to better manage their asthma symptoms and improve asthma-specific HRQoL.

One of the specific aims of the present study was to examine how parental health literacy, perceived self-efficacy with patient-physician interaction and satisfaction with SDM can contribute to children’s asthma control and asthma-specific HRQoL. Interestingly, we found a lack of association between health literacy and perceived self-efficacy with patient-physician interaction, which is consistent with a previous study [24], but in contrast to another study [23]. The reasons behind this lack of association may be confounded due to the increased self-confidence in parents who have established a long-term open and trusting relationship with their physicians [39]. On the other hand, it is possible that perceived self-efficacy with patient-physician interaction may be explained by personality traits (e.g., optimism), which is not influenced by one’s level of health literacy. Lastly, we had very few parents with low health literacy and that may have limited our ability to detect small, but important relationships between health literacy and perceived self-efficacy with patient-physician interaction.

Evidence is limited about the relationship between health literacy and satisfaction with SDM. Some studies reported that patients with lower health literacy were less likely to take part in the medical decision-making process [22,40]. The present study extends the previous findings, showing that parents with higher levels of health literacy had greater satisfaction with SDM. It is plausible that parents with high literacy levels were likely to take an active role and intensively engage in the shared-decision process, leading to an increase in their satisfaction with SDM. Designing appropriate interventions to improve health literacy levels, particularly asthma-relevant literacy, may foster the patient-physician communication, and increase the involvement of parents in the SDM [22].

The association between self-efficacy and asthma outcomes, especially asthma control and HRQoL, remains unclear. While the present study found that perceived self-efficacy was not directly associated with asthma control and asthma-specific HRQoL, others have previously noted this association [25,27]. From a design perspective, the discrepant findings may be due to the fact that previous studies investigated the influence of self-efficacy as part of the psychosocial coping resource [41] or as self-efficacy on HRQoL [27]; instead, our study tested the influence of perceived self-efficacy with patient-physician interaction on both asthma control and asthma-specific HRQoL.

This study identified several important pathways involved in good asthma control and asthma-specific HRQoL. The findings highlight the need for appropriate interventions to improve asthma control and asthma-specific HRQoL in children through the needs and strengths of their parents. Higher perceived self-efficacy with patient-physician interaction would be indirectly associated with good asthma control through the satisfaction with shared decision-making. This implies that if clinicians can improve the patient-doctor interactions through assuring that patients understand the asthma treatment plan and are satisfied with the interaction process, the likelihood of achieving good asthma control is high. On the other hand, higher perceived self-efficacy was indirectly associated with better asthma-specific HRQoL through satisfaction with SDM and asthma control. It thus seems appropriate that interventions targeted at increasing perceived self-efficacy and/or satisfaction with SDM will increase the likelihood of improving asthma outcomes.

The study is not without limitations. We utilized a cross-sectional study design to investigate the interrelationships among variables. This limits our ability to interpret the causal relationships among these variables. Our path analysis did, however, allow us to establish which of the potential mediator variables were most important in explaining the overall association with the asthma outcomes. The path analysis that we used in this study provides evidence whether the observed data were consistent with a priori hypotheses based on evidence available from several studies. We recognize that the causality in cross-sectional studies can only be speculated and be accepted with caution; longitudinal studies are needed to examine those associations. Second, participants were recruited from five pediatric clinics of a single university medical center, which may limit the generalizability of these findings to other populations. Third, we did not distinguish the role of parental health literacy on asthma-specific outcomes in children compared to adolescents. Oftentimes adolescents are more mature, have different cognitive abilities and are more responsible than children which may potentially influence their role in the decision making process [42]. Future studies should investigate the role of health literacy in adolescents and its relationship with self-efficacy, satisfaction with SDM, and asthma outcomes and how it differs to the role of parental health literacy in children for the said relationships. Fourth, we rely on parent report to collect child’s asthma control status because our clinical experience informs us that parents would better understand and recognize the types of medication than children did. Nevertheless, previous studies have shown that the discrepancy in parent and child reports were not different [43,44]; and parents and children tend to overstate asthma medication adherence compared to the use of asthma inhaler canister weight checks [44] and electronic canister measures that recorded daily adherence through a microchip [43]. More research is needed to test the accuracy and discrepancy in child self- or parent proxy-report of the medication use. Lastly, the small number of respondents may have influenced the model fit in the path analyses. Nevertheless, we used procedures noted to optimize use of SEM procedures in studies with small sample sizes [36,45].

## Conclusion

Children’s asthma control status influenced their asthma-specific HRQoL. However, several parental factors contributing to asthma control indirectly affected asthma-specific HRQoL. Parents with greater perceived self-efficacy with patient-physician interaction were more likely to be satisfied with SDM, which in turn was associated with better asthma control, leading to better asthma-specific HRQoL. Intervention studies focusing on improving self-efficacy and satisfaction with SDM are important to pursue for better improving asthma control and ultimately asthma-specific HRQoL.

## Abbreviations

CI: Confidence interval;HRQoL: Health-related quality of life;NAEPP: National asthma education and prevention program;NIH: National institute of health;PROMIS: Patient-reported outcomes measurement information system;RMSEA: Root mean square error of approximation;SDM: Shared decision-making;SEM: Structural equation modeling;S-TOFHLA: Short test of functional health literacy in adults;UF: University of Florida;χ2: Chi-square;λ: Factor loading

## Competing interests

The authors declare that they have no competing interests.

## Authors’ contributions

DAD, DAR, EAS, and ICH designed the study. KMK and LAT conducted data collection. PKG, KMK, and ICH analyzed the data and interpreted the results. PKG, KMK, and ICH wrote the manuscript. LAT, DAD, DAR, and EAS critically revised the manuscript. EAS supervised the entire study. All authors read and approved the final manuscript.
